# Effectiveness of motivational interviewing on improving Care for Patients with type 2 diabetes in China: A randomized controlled trial

**DOI:** 10.1186/s12913-019-4776-8

**Published:** 2020-01-23

**Authors:** Zhe Li, Qingqi Chen, Jingya Yan, Wei Liang, William C. W. Wong

**Affiliations:** 1 Family Medicine Department, The University of Hong Kong - Shenzhen Hospital, 1, Haiyuan 1st Road, Futian District, Shenzhen, China; 2 WHO Health Promoting Hospital Office, The University of Hong Kong - Shenzhen Hospital, Shenzhen, China; 3 Endocrinology Department, The University of Hong Kong - Shenzhen Hospital, Shenzhen, China; 4 Clinical Associate Professor, Department of Family Medicine and Primary Care, The University of Hong Kong, 1, Haiyuan 1st Road, Futian District, Shenzhen, China

**Keywords:** Type 2 diabetes, Motivational interviewing, Patient empowerment, Self-management, China

## Abstract

**Background:**

To assess the effects of a motivational interviewing (MI)-based patient empowerment program (PEP) on type 2 diabetes mellitus (DM) patient self-management compared to traditional diabetes health education.

**Methods:**

Two hundred and twenty-five patients, recruited from community health centers (CHCs) and the family medicine clinic in the University of Hong Kong-Shenzhen Hospital in Shenzhen, were randomly assigned to the intervention or control groups. Patients in the intervention group (*n* = 117) received a four-session PEP in small groups over 1 month by trained nurses and doctors. The control group (*n* = 108) received the traditional lecture-style health education on DM. All the patients were followed up for 3 months. Outcomes included problem areas in diabetes (PAID) that measures diabetes-related emotional distress, patient enablement index (PEI), mental health, patient satisfaction respectively as well as lifestyle behaviors were assessed at baseline, post-activity and 3 months.

**Results:**

At post-intervention and the 3-month follow-up, the PAID score improved significantly in the intervention group (12.7 ± 13.6, 5.8 ± 7.6) compared to the control group (22.7 ± 22.8, 11.7 ± 14.6). No difference was found between groups for changes to exercise, diet, and medication adherence. The PEI score improved significantly at the 3-month follow-up in the MI group (7.27 ± 2.45 vs 5.81 ± 2.97).

**Conclusion:**

The PEP has a significant effect on improving diabetes-related distress, but MI was not significantly different from the traditional health education programs when it comes to the readiness to change.

**Trial registration:**

NCT04120844, ClinicalTrials.Gov. Date of registration: October 9th 2019 (Retrospectively registered).

## Background

In 2015, the International Diabetes Federation estimated that there were nearly 110 million diabetes mellitus (DM) patients in China, which was the highest number recorded in the world. China’s DM-related costs, ranked second highest globally, were estimated to be US$51 billion [1]. In response to the rising patient numbers and costs, the Chinese government has invested heavily in primary healthcare since 2009, with the goal of improving chronic disease management in the primary care settings [2]. A key part of the primary care improvement program prioritizes health education as a route to lifestyle modification [3]. Although the content and modes of delivery vary enormously [4], most of the programs focused on providing information rather than facilitating patient change. The impacts of traditional patient education on lifestyle modification and changes in psychological status have been reported to be suboptimal [5]. These may be related to the poor understanding of the educational content or lack of means for making changes as a result of low socioeconomic status and poor educational level. It is therefore necessary to rethink and explore a more structured, patient-centered approach to health education at improving the outcomes of DM control. In one study, patients’ active involvement in their treatment decision-making process was positively related to their treatment goals [6].

Motivational interviewing (MI) is a collaborative, patient-centered counseling approach that aims to elicit behavior change [7]. Counselors use empathy and other techniques to create an atmosphere to help patients to explore the discrepancies between the goals and their current behavior. The focus of MI is to find and resolve the ambivalence, improve patients’ perception of the importance of behavior change, and support them to make the change. MI provides a structural framework with guiding principles that can be easily followed by the primary care doctors.

In a recent systematic review of MI, it was found that there were significant improvements in a number of the patient outcomes such as total cholesterol, fasting blood glucose, body mass index, blood pressure, waist circumference and physical activity [8]. In another study, HbA1c was reduced by as much as 1% with a single brief intervention followed by a short education session [9], and the effects can be sustained at 3 and 6 months after MI intervention [10]. Some studies show that MI can contribute to improve healthy eating, weight control [11, 12] and increases in physical activity [13], but most research focused on intermediate outcome measures [14] but did not evaluate the readiness to change. Ekong and colleagues found no studies had evaluated the impact of MI on medication-taking behaviors, which should be an important target behavior of DM management [11].

MI can be utilized by a variety of healthcare providers, which makes it adaptable for different culture and clinical settings [15]. However, the effectiveness of MI in Chinese diabetic patients remains uncertain. In the more recent Chinese literature, Chen and colleagues found MI significantly improved participants’ self-management, self-efficacy, quality of life and HbA1c level, but there were no changes in depression, anxiety and stress [16]. However, in another study by Browning and colleagues, it was found that MI had no differential treatment effect for HbA1c [17].

MI has been delivered using different methods. These methods have varied and included a single one-to-one session with a therapist, multiple group sessions, and the incorporation of MI into daily clinical practice. However, in one study, no statistically significant differences were found between individual and group delivery modes [18]. Furthermore, in yet another study, MI education program produced a significantly greater change in patients’ perceived competence in dealing with diabetes than the control group [19]. In this study, we adopted the group MI approach and developed a patient empowerment program (PEP) utilizing the techniques and framework of MI.

We compared this to the most common form of DM education in China, i.e., when health professionals (nurses, doctors, dietitians or pharmacists) give a lecture on DM to patients and their carers in a hospital lecture theatre in a didactic manner [5]. It was aimed to assess the effectiveness of the MI approach in terms of patient lifestyle modification and improving DM controls compared to the control group in a non-blinded randomized controlled trial (RCT) design.

## Methods

### Study design and participants

Before this RCT, a pilot study was conducted which tested the acceptability and feasibility of this study protocol. Recruitment resulted in 28 participants, with 26 completing all data collections and reporting improved mood.

This RCT was implemented from May 2016 to April 2017 in Shenzhen, China. Shenzhen is the fourth-biggest city in China, with a rapidly expanding population of 12 million people and the highest GDP in the country. Most DM patients in China are managed at Endocrine Specialist hospital clinics, while the more stable patients are treated in the community. Therefore, we chose an endocrine specialist outpatient clinic and a family medicine clinic at the University of Hong Kong-Shenzhen Hospital (HKU-SZH), as well as three community health centers in the Luohu district as the sampling frame. DM patients were recruited by doctors at consultations. This is because health education lectures are one of the routine diabetes management strategies used by healthcare providers in China and patients are normally invited by their doctors to attend these lectures.

**Fig. 1:**
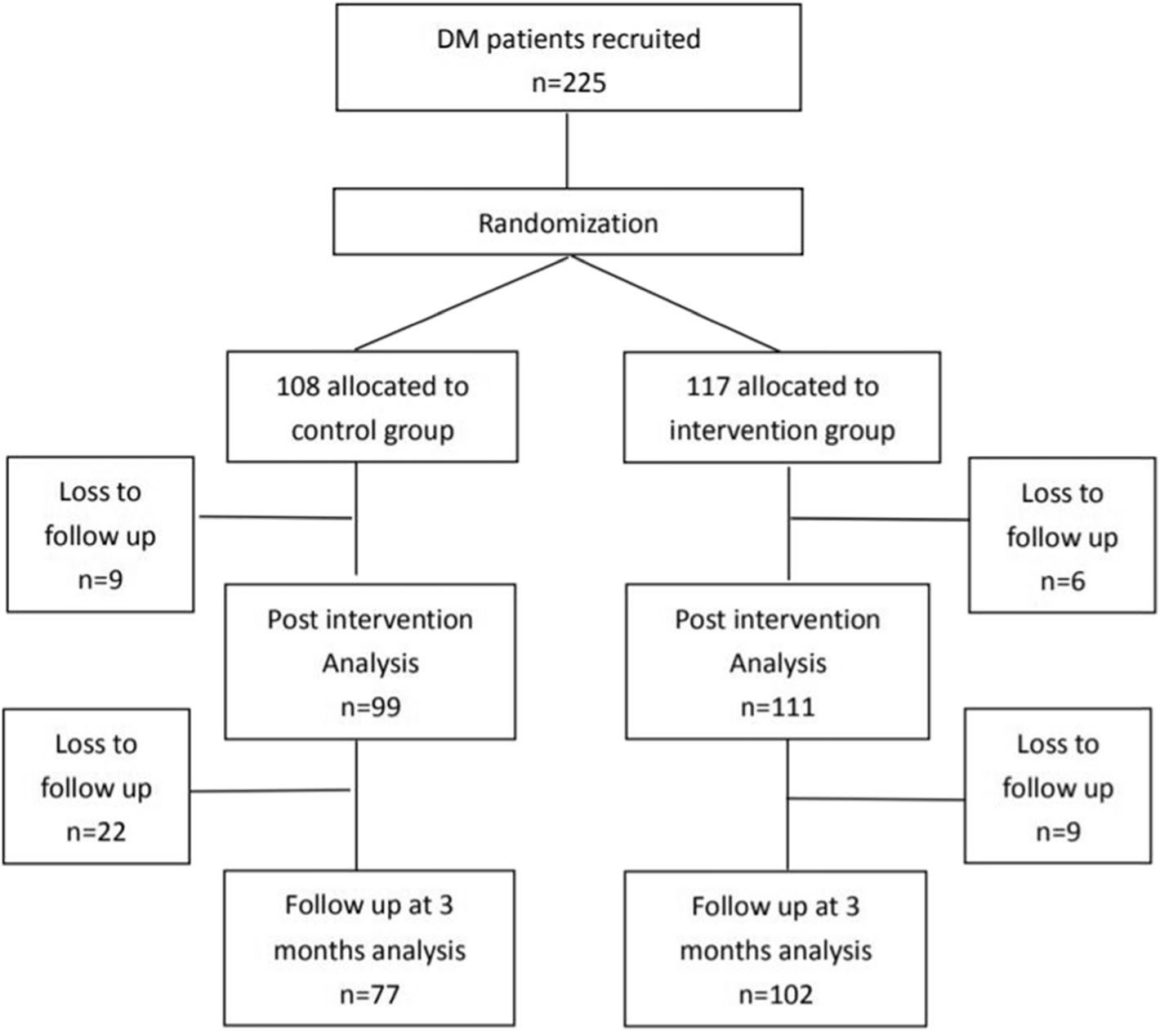
Flow diagram on recruitment and participation in this study (*n* = 225). How was the recruitment and participation?

The inclusion criteria were: 1. Type 2 diabetes with HbA1c between 7 and 10%; 2. 18–75 years old; 3. No known severe comorbidities or complications, such as cancer, unstable angina, frequent exacerbation of chronic obstructive pulmonary disease, or diabetic retinopathy; 4. Cognitively competent enough to understand written and the oral expression of the language native to the study site. Once the participants agreed to join, their names and contact information were sent to the research team for consent and randomization. (Fig. 1) They would be asked to fill in a pre-intervention questionnaire, immediately after the completion of all modules and another questionnaire at three-month follow-up. Participants could reschedule their appointment within 2 weeks if they were not able to attend the program.

The study and participant rights were explained to all participants before signing the written informed consent. Participation was voluntary and participants had the right to withdraw at any stage of the study. Patients were reassured that refusal to participate would not affect the provision of health services at the healthcare establishments.

### Sample size calculation

Sample size estimation was calculated based on the previous published research conducted elsewhere in which the DM patients were given MI-guided behavior change counselling. The “Problem Areas in Diabetes” (PAID) score in that study was 29 ± 22.64 in the intervention group vs. 29 ± 24.32 in the control group [20]. Therefore, 192 participants were needed to detect 10% effect size with an alpha of 0.05 and a power of 80.0%. With 15% of loss to follow-up anticipated, a total of 225 participants were targeted.

### Randomization

Upon receiving a participant’s consent form, the independent administrative research assistant would generate a random number by computer to determine allocation to the intervention or control group. For concealment purposes, there was no medical information relating to the participant on the consent form. The assistant only informed participants of their attendance time and not of their grouping. We used the blocked randomization by number “8” which guaranteed that there were four people in both groups in each of the eight people. This method can avoid overbalance between two groups as there might be some people missing halfway.

### Intervention

The intervention group received an education program in small groups that included no more than 10 members. The content was designed based on MI theory and the theory of patient empowerment [21]. Program content was further informed by the Hospital Authority Patient Empowerment Program in Hong Kong [22]. The education program consisted of four modules, held once a week, that each lasted approximately 1½ to 2 h. They were grouped under the following four broad headings: Knowing Diabetes, Diabetes Self-Care, Healthy Diet and Physical Exercise. Each module started with a brief introduction to relevant background knowledge, which was followed by small-group discussions about personal barriers and techniques for overcoming challenges. During the small group discussions, educators acted as MI facilitators, using group MI techniques to strengthen participants’ motivation. The main educator (Z.L) was a nurse who had attended a two-day workshop provided by the co-creator of MI, Dr. Stephen Rollnick. This workshop focused on the 4-process framework (Engage, Focus, Evoke, Plan) and common challenges experienced by patients, as well as interviewing strategies and techniques to use in the process. The nurse educator (Z.L) also received 1 week of supervision by an experienced primary care doctor who was trained by Prof. Richard Botelho *who is the author of a book “Motivational Practice” in promoting healthy habits and self-care of chronic disease*. The other educator team members were all supervised by the nurse educator (Z.L) and the experienced primary care doctor (W.W). Educators were also provided with a manual designed by the research team. The manual contained structured, semi-open questions to guide the educators through the small group discussions.

### Control group

The control group received traditional lectures that consisted solely of conveying healthcare information to patients. In order to minimize intervention bias, the control group lectures were standardized and adapted into four modules, namely knowing diabetes, healthy diet, physical exercises, and how to use medication correctly, which were similar topic headings, durations and frequencies to those of the intervention group. Each lecture was 1 h and was provided by one of four health professionals (a pharmacist, dietician, endocrinologist or nurse) who had never received any prior training in MI. We consciously avoided the inclusion of elements of self-reflection and motivation in these lectures.

### Outcome measures

The primary outcome measure in this study was the PAID score. The secondary outcome were the “Patient Enablement Index” (PEI) score and Stages of Change score. The PAID scale has been widely used in many countries to assess diabetes-related emotional distress [23]. Validated Chinese-language versions of PAID include both a 20-item Chinese version of PAID (PAID-C) [24], and an 8-item short-form PAID (SF-PAID-C) that were developed and validated in Taiwan [25]. This study used SF-PAID-C to assess diabetes-related emotional distress. The PEI is a scale that measures patients’ enablement, it was also used to measure patient enablement in this study. The PEI scale had been validated in the Chinese population [26].

Motivation for lifestyle change was measured in this study based on the “Stages of Change” model to assess participants’ readiness to change in behaviors such as smoking, drinking or exercise and their adherence to treatment. There are five stages in this model, including Precontemplation, Contemplation, Preparation, Action and Maintenance. Other clinical data were also collected including: body mass index (BMI), waist circumference, blood pressure (BP), etc. Both height and weight were carefully measured without a participant’s shoes, cell phone, keys or wallet. Waist circumference was measured with a measuring tape directly on the individual’s skin and placed at the belly button horizontal to the floor. BP was measured by automated BP machines and was taken at sitting position after participants rested for 10–15 min.

All the participants’ clinical data, as described above, were collected at baseline, as were their PAID scores. The PAID and PEI scores as well as Stages of Change were then assessed at post-intervention and 3-month follow-up.

### Statistical analysis

Descriptive statistics were used to summarize characteristics of the participants. We analyzed the baseline data of the intervention and control groups to determine the consistency of the characteristics across the two groups of patients. The *t*-test was used for continuous variables such as waist circumference, body weight, and BMI, whereas *chi*-squared test was used for categorical variables in stages of change such as smoking, drinking, and exercise. Changes in PAID and PEI in post-intervention and follow-ups between the two groups were calculated and tested.

When analyzing the two sets of variables in the intervention and control groups, we followed the principle of intent-to-treat analysis i.e. if the participant failed to participate in all four modules, the first questionnaire results would be assumed and analyzed as the final data, using the mixed design analysis of variance. In detecting the relationship between the continuity variable and categorical variable, we used bivariate correlation analyses. All analyses were performed in SPSS 20.0. We used *p* value< 0.05 as the cut-off point of statistical significance.

## Results

Figure 1 depicts the flow of participants through the trial. Two hundred and twenty five patients were randomized to the two groups, with *n* = 117 receiving MI and *n* = 108 in the control group. In the MI group and control group, 6 (5.1%) and 9 (8.3%) participants did not finish the program. With regard to the remaining 210 participants (MI: *n* = 111; control: *n* = 99), 9 (8.1%) and 22 (22.2%) participants in the MI group and control group were loss to the 3-month follow up. As a result, 102 and 77 participants in the MI group and control group were included in the 3-month follow up analysis.

The baseline characteristics on demographics, vital signs and lifestyle behaviors were largely similar between the two groups. Except the MI group were younger (57.4 ± 13.4 years vs. 61.9 ± 12.4 years, *p* = 0.01), and the diastolic blood pressure were lower in the MI group (73.7 ± 9.9 mmHg vs 78.2 ± 9.5 mmHg, *p* = 0.00). The duration of type 2 DM in the MI group appeared to be shorter (6.8 ± 5.7 years vs 7.9 ± 6.7 years, *p* = 0.22), with a higher proportion of this group having no comorbidities (34.2% vs. 16.7%). There were more women (50.7%) than men in the sample. The majority were unemployed (61.8%) and had received secondary or higher education (65.8%). The smoking rate in the sample was 10.7, and 15.1% of the participants were classified as drinker. (Table 1).

**Table 1:** Participant demographic, medical history, and lifestyle behavior characteristics (*N* = 225)

Demographic Variable	Intervention	Control	Total	*p*
	*n* = 117 (52%)	*n* = 108(48%)	N = 225	
**Age (mean ±** ***SD*)**	57.4 ± 13.4	61.9 ± 12.4	59.6 ± 13.1	0.01*
**Gender (*n*, %)**				0.85
Male	60(51.3%)	54(50%)	114(50.7%)	
Female	57(48.7%)	54(50%)	111(49.3%)	
**Occupation (*n*, %)**				0.45
Full time	26(22.2%)	19(17.6%)	45(20%)	
Part time	3(2.6%)	6(5.6%)	9(4%)	
Unemployed	65(55.6%)	74(68.5%)	139(61.8%)	
Self-employed	8(6.8%)	2(1.9%)	10(4.4%)	
Others	15(12.8%)	7(6.4%)	22(9.8%)	
**Education Level (*n*, %)**				0.65
No or primary	41(35.0%)	36(33.4%)	77(34.2%)	
Secondary	47(40.2%)	52(58.1%)	99(44%)	
Tertiary	29(24.8%)	20(18.5%)	49(21.8%)	
**Duration of T2DM in years (mean ±** ***SD*)**	6.8 ± 5.7	7.9 ± 6.7	7.3 ± 6.2	0.22
**BP (mean ±** ***SD*)**				
Systolic BP(mmHg)	122.8 ± 16.8	125.7 ± 17.3	124.2 ± 17.1	0.21
Diastolic BP(mmHg)	73.7 ± 9.9	78.2 ± 9.5	75.8 ± 10.0	0.00*
**BMI (mean ±** ***SD*)**	24.6 ± 3.6	24.2 ± 4.2	24.4 ± 3.9	0.39
**Waist (cm) (mean ±** ***SD*)**	87.7 ± 14.0	86.9 ± 10.8	87.3 ± 12.6	0.63
**PHQ-2 (mean ±** ***SD*)**	1.04 ± 1.69	0.97 ± 1.31	1.0 1 ± 1.52	0.73
**Comorbid conditions (*n*, %)**				0.15
None	40(34.2%)	18(16.7%)	58(25.8%)	0.05*
Cardiovascular disease	41(35.0%)	38(35.2%)	79(35.1%)	0.93
Non-Cardiovascular disease	36(30.8%)	52(48.1%)	88(39.1%)	0.14
**Smoking**				
Smoker (*n*, %)	11(9.4%)	13(12.0%)	24(10.7%)	0.52
Cigarettes per day (mean ± *SD*)	13.7 ± 9.5	8.7 ± 5.8	11.2 ± 8.1	0.13
**Alcohol**				
Drinker (*n*, %)	20(17.1%)	14(13.0%)	34(15.1%)	0.39
Units per day (mean ± *SD*)	1.7 ± 1.9	2.7 ± 3.2	2.2 ± 2.7	0.30
**Exercise (Moderate to Intensive)**				0.40
Less than 15 days per month (*n*, %)	60(51.3%)	41(38.0%)	101(44.9%)	0.07
15 days per month or above (*n*, %)	57(48.7%)	67(62.0%)	124(55.1%)	0.12

At baseline, the PAID scores were very similar across the intervened and control groups. The exception to this similarity was their ability to manage complications of DM. Here, the mean in the control group was higher (1.33 in the control vs. 0.78 in the intervened; *p* = 0.02). (Table 2) PAID scores within groups were significantly improved at post-intervention and 3-month follow-ups in the intervened group but only at 3-month follow-ups in the control group. Compared to the control group, all items of the PAID scores were significantly improved in the intervention groups at both post-intervention and 3-month follow-ups.

**Table 2:** PAID scores between intervention and control at baseline, post-intervention and 3-month follow-up

	Intervention(*n* = 117)			Control(*n* = 108)			Intervention vs Control		
	Baseline	Post-intervention	Follow-up	Baseline	Post-intervention	Follow-up	Baseline	Post-intervention	Follow-up
	Mean (*SD*)	Mean (*SD*)	Mean (*SD*)	Mean (*SD*)	Mean (*SD*)	Mean (*SD*)	*P value*	*P value*	*P value*
1. Feeling scared when you think about living with diabetes	1.21(1.18)	0.70(0.91)*	0.24(0.53)*	1.33(1.27)	1.13(1.24)	0.58(0.86)*	0.43	0.01	0.00
2. Feeling depressed when you think about living with diabetes	1.09(1.13)	0.60(0.91)*	0.21(0.54)*	1.23(1.27)	1.03(1.20)	0.49(0.82)*	0.36	0.00	0.01
3. Feeling overwhelmed by your diabetes regimen	0.60(1.01)	0.32(0.71)*	0.12(0.40)*	0.86(1.16)	0.84(1.23)	0.37(0.75)*	0.08	0.00	0.00
4. Feeling guilty or anxious when you get off-track with your diabetes management	0.85(0.99)	0.55(0.87)*	0.20(0.45)*	1.04(1.21)	1.15(1.25)	0.49(0.80)*	0.21	0.00	0.00
5. Coping with complications of diabetes	0.78(1.07)	0.51(0.92)*	0.15(0.45)*	1.33(1.47)	1.21(1.39)	0.46(0.78)*	0.00	0.00	0.00
6. Feeling that diabetes is taking up too much mental and physical energy	0.79(1.05)	0.47(0.82)*	0.21(0.51)*	0.96(1.28)	1.10(1.30)	0.49(0.75)*	0.27	0.00	0.00
7. Feelings of deprivation regarding food and meals	1.34(1.04)	0.96(0.81)*	0.57(0.64)*	1.53(1.42)	1.36(1.31)	0.84(0.87)*	0.26	0.01	0.02
8. Feeling constantly concerned about food and eating	1.39(1.01)	0.96(0.85)*	0.58(0.62)*	1.42(1.32)	1.21(1.26)	0.80(0.89)*	0.84	0.09	0.06
**Total** (adjusted)	**20.1(16.9)**	**12.7(13.6)**	**5.8(7.6)**	**24.4(22.4)**	**22.7(22.8)**	11.7(14.6)*	**0.17**	**0.00**	**0.00**

* *p* < 0.05

It appears that, of all items in both intervention and control groups PEI scores were positively improved at post-intervention (0.63–0.86 in the intervention vs. 0.75–0.90 in the control). (Table 3) However, at 3-month follow-ups, only sustained improvements in all PEI items were observed in the intervention group. In the control group in the same period, only the item, “can understand my illness” showed a sustained improvement (0.95 in the control vs. 1.19 in the intervention). When compared to the control group, a statistically significant difference in the response of “can keep own health” was observed (0.81 in the control vs. 0.61 in the intervention). However, the intervention group demonstrated significant improvements at 3-month follow-ups in all items of the PEI, when compared to those of the control group.

**Table 3:** PEI scores between intervention and control at post-intervention and 3-month follow-up

	Intervention(*n* = 117)		Control(*n* = 108)		Intervention vs Control	
	Post-intervention	Follow-up	Post-intervention	Follow-up	Post-intervention	Follow-up
	Mean (*SD*)	Mean (*SD*)	Mean (*SD*)	Mean (*SD*)	*P value*	*P value*
1. Able to face your life	0.63(0.64)	1.14(0.53)*	0.81(0.70)	0.96(0.57)	0.06	0.03
2. Able to understand your illness	0.86(076)	1.19(0.54)*	0.75(0.68)	0.95(0.58)*	0.31	0.01
3. Able to live with your illness	0.68(0.65)	1.18(0.53)*	0.75(0.74)	0.92(0.58)	0.44	0.00
4. Able to maintain good health	0.61(0.65)	1.19(0.50)*	0.81(0.74)	0.96(0.54)	0.03	0.00
5. Confident about your health	0.71(0.66)	1.30(0.48)*	0.89(0.76)	1.00(0.58)	0.08	0.00
6. Able to self-help	0.70(0.67)	1.28(0.48)*	0.90(0.71)	0.95(0.58)	0.07	0.00
**Total**	4.2(3.23)	7.27(2.45)	4.97(3.73)	5.81(2.97)	0.12	0.00

* p < 0.05

All stages of change scores were high (i.e. > 3 out of four) in both intervention and control groups. Some changes in readiness to exercise (3.54 in the control vs. 3.34 in the intervention) were reported at 3-month follow-ups for both the intervention and control groups. However, there were no statistical differences in self-reported lifestyle changes, including exercise, diet, and adherence to treatment, within group and between groups at post-intervention and at the 3-month follow-ups. (Table 4).

**Table 4:** Stages of Change scores between intervention and control at post-intervention and 3-month follow-up

	Intervention(*n* = 117)				Control(*n* = 108)				Intervention vs Control	
	Post-intervention		Follow-up		Post-intervention		Follow-up		Post-intervention	Follow-up
	Mean (*SD*)	Median	Mean (*SD*)	Median	Mean (*SD*)	Median	Mean (*SD*)	Median	*P value*	*P value*
1. Exercise	3.07(0.97)	3	3.34(0.84)*	4	3.22(1.02)	4	3.54(0.80)*	4	0.27	0.10
2. Diet	3.08(0.98)	3	3.30(0.88)	3	3.27(1.01)	4	3.53(0.85)	4	0.17	0.08
3. Medication adherence	3.05(1.12)	3	3.07(1.20)	3	3.00(1.34)	3	3.23(1.15)	4	0.79	0.46

Stages of Change scores: 0, pre-contemplation; 1, contemplation; 2, preparation; 3, action; 4, maintenance

* p < 0.05

## Discussion

Two hundred and twenty-five patients, recruited from CHCs and the family medicine clinic at the University of Hong Kong-Shenzhen Hospital in Shenzhen, were randomly assigned to the intervention group (*n* = 117) that received MI-based group PEP over four sessions, or the control group (*n* = 108) that received the traditional lecture-style health education on DM. The baseline characteristics were largely similar in both groups, but the statistics analyses showed that the intervention group was younger, and had fewer years of DM and fewer comorbidities, which mean they could have a better understanding of the workshop content as previously studies found socioeconomic factors and educational level have significant roles to play [5]. Although the dropout rates of the two groups were low (5.1 and 8.3%), there were significantly more (22 participants) in the control group were lost follow up at the 3-month survey, which can be interpreted as meaning that the control group was less engaged. This study found that MI improved the DM patients’ PAID and PEI scores significantly compared to traditional approach of lecture-style patient education. These effects were even more obvious at the 3-month follow-ups, demonstrating a delayed but profound effect of MI on patients’ empowerment and efficacy in these difficult areas of care. However, there was no difference in the readiness of lifestyle changes i.e. exercise, diet and medication adherence. It was hoped that changes on the relevant behaviors will eventually impact clinical outcomes, but this takes time and would be much more difficult to achieve since they tend to be affected by multiple variables than those collected in this study and may take a larger sample size to witness the chnages.

Our study found that the MI group scored much higher than the control group on the PAID scale, suggesting that the MI approach has an advantage over the traditional lectures in effectiveness at addressing patients’ perceived problematic areas. At the 3-month follow-up analyses, both PEI and PAID scores in the MI group were superior to those of the control group, suggesting that MI was more effective and persistent with patient empowerment and distress improvement than the control group educational program. Our findings are consistent with the recent systematic review by Knight [27], in which MI was found to have had positive effects on psychological outcomes of DM patients. However, in that review, there was one MI study delivered by a psychologist in which significant weight loss in type 2 diabetic women was shown [28]. It is, therefore, possible for MI to improve diabetic patients’ psychological condition but further study is needed.

Both the PAID and PEI score increases were assessed at the 3-month follow-ups, rather than at post-intervention analysis. This may be related to the relative short follow-up period, as some patients were still learning and applying their new skills. The use of new knowledge and skills may bring more confidence to the patient, thereby reducing stress at the follow-ups.

The Stages of Change score was greater than 3 in both groups after intervention and at the 3-month follow-ups, indicating that our patients in both groups were more prepared to change, and that their readiness to change could be sustained in the studied period. In our study, the MI group did not show any advantage over the control in lifestyle change readiness. This may be because DM is a complex, multifaceted metabolic disease requiring long-term and consistent management in order for them, taken into the consideration of the environment in which people live and work, in order for them to commit to a lifestyle change to achieve optimal control [29]. One of the criticisms in the literature is that researchers often do not provide adequate information on the quality and intensity of the intervention and fidelity to the MI approach, so one does not really know whether MI is ineffective or if something else might have inhibited its possible impact. Nonetheless, many researchers have shown the positive effects of MI in managing DM, such as the role of MI training [30, 31], the role of nursing staff in self-management and quality of life [16] and the effects on behavior [11, 32].

The key strength of this research is that the MI PEP approaches health education as closely as possible to the existing health education model in China, maximizing its external validity. According to Chinese policy [33], all health care providers must provide health education. This study followed the same approach used by health care providers to recruit patients and provide patient education. The patients who participated in the intervention group did not receive more education time than those in the control group, nor did they receive other care. The only difference between the MI and control groups was the content and patient approach. The majority of time was spent on improving motivation in the MI group whilst the control group focused on providing information. Some of the limitations of this study are: the relatively short follow-up times, the lack of intervention fidelity assessment which might have contributed to minimal effects of MI on some target variables, and the absence of objective indicator measures, such as home blood sugar monitoring and glycated hemoglobin (HbA1c). There is obviously a risk of over-reporting by the patients due to social desirability bias. However, the choice of measures was deliberate: some of the previous studies on MI have not shown a clear role in improving HbA1c [14, 17], and this study therefore aimed to explore whether MI can improve the patient’s willingness to change.

## Conclusion

The Chinese government has created the primary care and is looking for new approaches to cope with an increasingly large number of DM patients. This study examined the effectiveness of group MI-based PEP over traditional classes to improve DM control. Our study found that MI had significant effects in improving DM-related distress and improved efficacy (as shown in the PAID and PEI results) immediately post-intervention and at 3-month follow-up, but not in the readiness for behavioral change. Peer interactions in group MI has the advantage of generating psychosocial support from people facing similar challenges in making changes in a similar environment or context, role modelling and group problem-solving. It is believed that this support and individualized behavioral skills beyond health information, as advocated in MI, are the first steps to improving DM control in the long term.

## Data Availability

The datesets used and analysed during the current study are available from the corresponding author on reasonable request.

## Abbreviations

BMI: Body mass index
CHCs: Community health centers
DM: Diabetes mellitus
HbA1c: Glycated hemoglobin
HKU-SZH: Hong Kong-Shenzhen Hospital
MI: Motivational interviewing
PAID: Problem areas in diabetes
PEI: Patient enablement index
PEP: Patient empowerment program
RCT: Randomized controlled trial

## References

[1] International Diabetes Federation (2015). IDF diabetes atlas.

[2] Liu Q, Wang B, Kong Y, Cheng KK (2011). China's primary health-care reform. Lancet.

[3] Chinese Diabetes Society (2014). China’s prevention and treatment guideline for type 2 diabetes mellitus (2013 edition). Chin J Diab Mellitus.

[4] Lou Q, Wu L, Dai X, Cao M, Ruan Y (2011). Diabetes education in mainland China—a systematic review of the literature. Patient Educ Couns.

[5] Guo XH, Yuan L, Lou QQ, Shen L, Sun ZL, Zhao F, Dai X, Huang J, Yang HY (2012). A nationwide survey of diabetes education, self-management and glycemic control in patients with type 2 diabetes in China. Chin Med J.

[6] Cornell S (2013). A patient-centred approach to treatment with incretin-based agents in patients with type 2 diabetes. J Clin Pharm Ther.

[7] Miller WR, Rollnick S. Motivational interviewing: preparing people for change. 2nd ed: New York, Guilford Press; 2002.

[8] Thepwongsa I, Muthukumar R, Kessomboon P (2017). Motivational interviewing by general practitioners for type 2 diabetes patients: a systematic review. Fam Pract.

[9] Wang YC, Stewart SM, Mackenzie M (2010). A randomized controlled trial comparing motivational interviewing in education to structured diabetes education in teens with type 1 diabetes. Diabetes Care.

[10] Song Dan, Xu Tu-Zhen, Sun Qiu-Hua (2014). Effect of motivational interviewing on self-management in patients with type 2 diabetes mellitus: A meta-analysis. International Journal of Nursing Sciences.

[11] Ekong G, Kavookjian J (2016). Motivational interviewing and outcomes in adults with type 2 diabetes: a systematic review. Patient Educ Couns.

[12] Lundahl B, Moleni T, Burke BL, Butters R, Tollefson D, Butler C, Rollnick S (2013). Motivational interviewing in medical care settings: a systematic review and meta-analysis of randomized controlled trials. Patient Educ Couns.

[13] Martins RK, McNeil DW (2009). Review of motivational interviewing in promoting health behaviors. Clin Psychol Rev.

[14] Jansink R, Braspenning J, Keizer E, van der Weijden T, Elwyn G, Grol R (2013). No identifiable Hb1Ac or lifestyle change after a comprehensive diabetes programme including motivational interviewing: a cluster randomised trial. Scand J Prim Health Care.

[15] Emmons KM, Rollnick S (2001). Motivational interviewing in health care settings: opportunities and limitations. Am J Prev Med.

[16] Chen SM, Creedy D, Lin HS, Wollin J (2012). Effects of motivational interviewing intervention on self-management, psychological and glycemic outcomes in type 2 diabetes: a randomized controlled trial. Int J Nurs Stud.

[17] Browning C, Chapman A, Yang H, Liu S, Zhang T, Enticott JC, Thomas SA (2016). Management of type 2 diabetes in China: the happy life Club, a pragmatic cluster randomised controlled trial using health coaches. BMJ Open.

[18] Lundahl BW, Kunz C, Brownell C, Tollefson D, Burke BL (2010). A meta-analysis of motivational interviewing: twenty-five years of empirical studies. Res Soc Work Pract.

[19] Rosenbek Minet LK, Wagner L, Lønvig EM (2011). The effect of motivational interviewing on glycaemic control and perceived competence of diabetes self-management in patients with type 1 and type 2 diabetes mellitus after attending a group education programme: a randomosed controlled trail. Daibetologia.

[20] Gabbay RA, Añel-Tiangco RM, Dellasega C, Mauger DT, Adelman A, Van Horn DHA (2013). Diabetes nurse case management and motivational interviewing for change (DYNAMIC): results of a 2-year randomized controlled pragmatic trial. J Diab.

[21] Tang TS, Funnell MM, Brown MB, Kurlander JE (2010). Self-management support in “real-world” settings: an empowerment-based intervention. Patient Educ Couns.

[22] Wong CK, Wong WC, Lam CL, Wan YF, Wong WH, Chung KL (2014). Effects of patient empowerment Programme (PEP) on clinical outcomes and health service utilization in type 2 diabetes mellitus in primary care: an observational matched cohort study. PLoS One.

[23] Polonsky WH, Anderson BJ, Lohrer PA, Welch G, Jacobson AM, Aponte JE, Schwartz CE (1995). Assessment of diabetes-related distress. Diabetes Care.

[24] Huang MF, Courtney M, Edwards H, McDowell J (2010). Validation of the Chinese version of the problem areas in diabetes (PAID-C) scale. Diabetes Care.

[25] Hsu HC, Chang YH, Lee PJ, Chen SY, Hsieh CH, Lee YJ, Wang RH (2013). Developing and psychometric testing of a short-form problem areas in diabetes scale in Chinese patients. J Nurs Res.

[26] Lam CL, Yuen NY, Mercer SW, Wong W (2010). A pilot study on the validity and reliability of the patient enablement instrument (PEI) in a Chinese population. Fam Pract.

[27] Knight KM, McGowan L, Dickens C, Bundy C (2006). A systematic review of motivational interviewing in physical health care settings. Br J Health Psychol.

[28] West DS, DiLillo V, Bursac Z (2007). Motivational interviewing improves weight loss in women with type 2 diabetes. Diabetes Care.

[29] Söderlund LL, Madson MB, Rubak S (2011). A systematic review of motivational interviewing training for general health care practitioners. Patient Educ Couns.

[30] Butler CC, Rollnick S, Cohen D (1999). Motivational consulting versus brief advice for smokers in general practice: a randomised trial. Br J Gen Pract.

[31] Welch G, Rose G, Ernst D (2006). Motivational interviewing and diabetes: what is it, how is it used, and does it work?. Diab Spect.

[32] DiClemente RJ, Crosby RA, Kegler MC (2002). Emerging theories in health promotion practice and research: strategies for improving public health.

[33] American Diabetes Association (2014). Diagnosis and classification of diabetes mellitus. Diab Care.

